# Supplementary material for: WordCommentsAnalyzer: A windows software tool for qualitative research
Source: F1000Res. 2018 Sep 4;7:536. Originally published 2018 May 3. [Version 2] doi: 10.12688/f1000research.14819.2 (PMC6137411; doi:10.12688/f1000research.14819.2)
Supplement: Supplementary file 3 [file f1000research-7-17614-s0002.tgz › b2a79496-4ada-47c4-9682-a99ce867836d.zip!/Eight Iranian Health Journals/Journal of Knowledge and Health_11.docx]

© Journal of Knowledge and Health. This work is licensed under CC BY 4.0 (https://creativecommons.org/licenses/by/4.0/).

Volume & Issue: دوره 10، شماره 3: 1394

Title: تأثير آرام‌سازی پیشرونده عضلانی بر سبک زندگی بیماران مبتلابه انفارکتوس قلبی: کارآزمایی بالینی تصادفی شده دو سویه کور

http://knh.shmu.ac.ir/index.php/site/article/view/635

مقدمه: امروزه بيماري‌هاي غيرواگير به‌دلایل نوسازي جوامع، پيشرفت فناوري و تراکم جميعت در مناطق شهري، تغيير سبک و شيوه زندگي، شيوع گسترده‌اي دارد. انواع بيماري‌هاي قلبي و عروقي شايع‌ترين علت مرگ در بيشتر کشورهاي جهان و ايران و مهمترين عامل از‌ کارافتادگي است. این پژوهش با هدف تعیین تأثير آرام‌سازی پیشرونده عضلانی بر سبک زندگی بیماران مبتلابه انفارکتوس قلبی انجام شد. مواد و روش‌ها: در این مطالعه کارآزمایی بالینی تصادفی دو سویه کور، 66 بیمار مورد پژوهش پس از نمونه‌گیری به‌طور تصادفی در دو گروه آزمون و شاهد قرار گرفتند. یک جلسه آشنایی‌سازی نمونه‌ها با اهداف و روش مداخله برگزار شد. گروه آزمون، روزانه دو جلسه20-30 دقیقه به مدت دو ماه (120 جلسه) تکنیک آرام‌سازی پیشرونده عضلانی را انجام دادند. ابزارهای جمع‌آوری اطلاعات شامل پرسشنامه اطلاعات دموگرافیک، پرسشنامه سبک زندگی و چک لیست خود گزارش‌دهی بود. نتایج: آزمون‌های تی مستقل و یو من ویتنی در چهار بعد سبک زندگی قبل از مداخله، تفاوت معنی‌داری را نشان ندادند اما این تفاوت بعد از مداخله معنی‌دار بود (05/0P<). همچنین آزمون ANOVA با اندازه‌های مکرر در سه زمان مختلف (قبل، 1 و 2 ماه بعد از مداخله) بین دو گروه آزمون و شاهد تفاوت معنی‌داری را نشان داد (05/0P<). نتیجه‌گیری: اجرای تکنیک آرام‌سازی پیشرونده عضلانی سبب اصلاح، تعدیل و بهبود ابعاد مختلف سبک زندگی بیماران مبتلابه انفارکتوس قلبی خواهد شد.

Title: بررسی تأثير تمرین مقاومتی بر سطوح عامل نوروتروفیک مشتق از مغز در پلاسمای موش‌های صحرایی

http://knh.shmu.ac.ir/index.php/site/article/view/679

مقدمه: عامل نوروتروفیک مشتق از مغز (BDNF) یکی از اعضای خانواده نوروتروفین‌ها است که نقش کلیدی در تنظیم بقا، رشد و حفظ نورون‌ها دارد.BDNF در پاسخ‌های سلولی و مولکولیبه تمرین مشارکت مي‌کند؛ اما آثار مداخلات تمرین برآن هنوز به‌خوبی درک نشده است. بنابراین، هدف این پژوهش، بررسی تأثير تمرین مقاومتی بر میزانعامل نوروتروفیک مشتق از مغز در پلاسمای موش‌های صحرایی بود. مواد و روش‌ها: 40 سر موش صحرايی ماده ویستار به‌طور تصادفی به 2 گروه شاهد و گروه تمرین مقاومتی تقسیم شدند. گروه تمرین مقاومتی شامل گروه یک جلسه تمرین و گروه 4 هفته تمرین بود. بالا رفتن از نردبان همراه با حمل وزنه به‌عنوان تمرین مقاومتی در نظرگرفته شد. هر جلسه تمرین شامل 3 نوبت با 5 تکرار بود. حیوانات، در گروه یک جلسه تمرین،0، 24، 48 و 72 ساعت و در گروه 4 هفته تمرین 24، 48 و 72 ساعت بعد از آخرين جلسه تمرين، بیهوش شدند و خونگیری انجام شد. نتایج: یافته‌های این پژوهش نشان داد که اختلاف سطوح  BDNFپلاسما بین دو گروه تمرینی یک جلسه و 4 هفته در مدت زمان 24 ساعت (0=P)، 48 ساعت (001/0=P) و 72 ساعت (002/0=P) معني‌دار است. سطوح BDNF پلاسما به‌دنبال 4 هفته تمرین در مدت زمان 24 و 72 ساعت پس از آخرین جلسه تمرین نسبت‌به گروه کنترل به‌طور معني‌داری كاهش يافت (به‌ترتیب 04/0 P=و 02/0P=). اما، به‌دنبال یک جلسه تمرین در مدت زمان بلافاصله، 24 و 72 ساعت، نسبت‌به گروه کنترل به‌طور معني‌داری افزایش يافت (به‌ترتیب 001/0=P، 009/0=P و 027/0=P). نتیجه‌گیری:تعداد جلسات تمرین مي‌تواند بر سطح نوروتروفین توليد شده در پاسخ به تمرین مقاومتی مؤثر باشد بنابراينتمرین مقاومتی يكي از مدل‌هاي مناسب و مؤثر برای بررسی رفتار مولکولی اين نوروتروفین است.

Title: تأثير ده هفته ورزش هوازی بر عملکرد سیستولیک بطن چپ، سطح کاسپاز-3 و اندازه ناحیه انفارکتوس موش‌های صحرایی با انفارکتوس قلبی

http://knh.shmu.ac.ir/index.php/site/article/view/680

مقدمه: هدف این مطالعه، بررسي تأثير ده هفته تمرین هوازی زیر بیشینه دویدن بر عملکرد سیستولیک بطن چپ، کاسپاز-3 و اندازه ناحیه انفارکتوس در موش‌های صحرايي با انفارکتوس قلبی می‌باشد. مواد و روش‌ها: 20 سر موش صحرایی نر ویستار چهار هفته بعد از انفارکتوس قلبی به دو گروه کنترل انفارکتوس شده (SedMI، 10n=) و گروه تمرینی انفارکتوس شده (ExMI، 10n=) تقسیم‌بندی شدند. 10 سر موش صحرايي نر ويستار بدون انفاركتوس قلبي نيز به‌عنوان گروه کنترل سالم (Sham، 10n=) در نظر گرفته شدند. گروه ExMI به مدت 10 هفته تمرین ورزشی دویدن بر روی تردمیل (هر هفته 5 جلسه و هر جلسه 50 دقیقه با سرعت 17 متر بر دقیقه معادل 55 درصد ظرفیت هوازی) را انجام دادند. از تمامي موش‌ها قبل و بعد از مداخله ورزش، اکوکاردیوگرافی به‌عمل آمد. نتايج : تجزيه و تحليل آماری داده‌ها نشان داد که میزان کسر تخلیه (EF) و کوتاه‌شدگی میوفیبریل‌های عضلانی (FS) در گروه‌های انفارکتوس شده (Ex MI ,Sed MI) نسبت‌به گروه شم ظرف 4 و 14 هفته بعد از انفارکتوس به‌طور معني‌داری کاهش داشت (05/0P<). میزان EF و FS در پایان هفته چهاردهم در گروه ExMI، بیشتر از گروه SedMI بود. همچنین اندازه ناحیه انفارکتوس و نرخ مرگ ‌و ‌میر جوندگان در پاسخ به فعالیت ورزشی به‌طور معني‌داری کاهش پیدا کرد (05/0P<)، اما تغییر معني‌داری در اندازه فعالیت کاسپاز-3 به‌دست نیامد (07/0P=). نتیجه‌گیری: به‌نظر می‌رسد که مداخله ده هفته ورزش هوازی متعاقب چهار هفته بعد از انفارکتوس قلبی موش‌ها، اختلالات عملکرد بطن چپ را کاهش می‌دهد و در نتیجه منجر به بهبود کارآیی سیستولیک بطن چپ، کاهش اندازه ناحیه انفارکتوس و نرخ مرگ و میر می‌شود.

Title: تأثير ماساژ پاها بر سطح هوشیاری بیماران کمایی ضربه مغزی بستری در بخش مراقبت‌های ویژه(ICU): یک مطالعه کارآزمایی بالینی

http://knh.shmu.ac.ir/index.php/site/article/view/692

مقدمه: کما از نتایج اصلی صدمه مغزی است که مي‌تواند به‌واسطه صدمات تروماتیک یا غیر تروماتیک عارض گردد. ماساژ پاها می‌تواند در تغییر سطح هوشیاری بیماران کمایی مؤثر باشد. این مطالعه با هدف تعیین تأثير ماساژ پاها بر سطح هوشیاری بیماران کمایی ضربه مغزی بستری در بخش مراقبت‌های ویژه (ICU) بیمارستان‌های منتخب شهر قزوین انجام شد. مواد و روش‌ها: این پژوهش از نوع كارآزمايي بالینی بوده که بر روی 40 بیمار کمایی بستری در بخش آی سی یو بیمارستان‌های شهید رجایی و تأمین اجتماعی شهر قزوین در سال 1393 انجام شد. بیماران با روش تخصیص تصادفی بلوکی در دو گروه مداخله و کنترل قرار گرفتند و سپس ماساژ هر دو پا به روش استروک (هر پا به مدت 5 دقیقه) برای 14 روز، روزانه یک نوبت انجام شده و سطح هوشیاری با استفاده از مقیاس کمای گلاسگو ثبت شد. جهت تجزیه وتحلیل داده‌ها از آزمون آماری (مجذور کای، تی مستقل، تی وابسته و آنالیز واریانس با اندازه‌های تکرار شونده) استفاده شد. نتایج:مطالعه، نشان مي‌دهد كه بين میانگین سطح هوشیاری در گروه مداخله قبل از شروع ماساژ (58/1±80/5) و بعد از انجام ماساژ (41/2±6/10) اختلاف آماری معنی‌داری وجودداشت (001/0P=)، اما بين میانگین سطح هوشیاری در گروه کنترل قبل از مداخله (72/1±30/5) و پس از مداخله (03/3±94/6) طی 14روز اختلاف آماري معناداري وجود نداشت (06/0P=). نتیجه‌گیری: ماساژ پاها می‌تواند سبب افزایش سطح هوشیاری بیماران کمایی ضربه مغزی شود و به‌عنوان روشی جهت افزایش سطح هوشیاری بیماران کمایی پیشنهاد می‌شود.

Title: بررسي اثر جريان متناوب بر روي توانایی تشکیل بیوفیلم باکتري‌هاي پسودوموناس آئروژينوزا و استافيلوکوک اورئوس

http://knh.shmu.ac.ir/index.php/site/article/view/704

مقدمه: بيوفيلم‌هاي باكتريال يكي از مشكلات اصلي ضدعفوني كردن مراكز بهداشتي است. مطالعات نشان مي‌دهد كه اثر جریان مستقیم بر روی بیوفیلم باعث بهبود کارآيي عوامل ضد باکتريايي بر عليه بيوفيلم مي‌شود. اما مطالعات کمی در مورد آثار بیوالکتریک جریان متناوب بر روی رشد باكتري و احتمال تشكيل بیوفیلم انجام شده است. لذا در مطالعه حاضر اثر جريان‌هاي الکتريکي متناوب با فرکانس‌ها و ولتاژهای مختلف بر روي توانایی تشکيل بيوفيلم باکتری ارزیابی شد. مواد و روش‌ها: در اين مطالعه، اثر جريان متناوب با فرکانس‌های مختلف (1، 5، 10 و 20 مگاهرتز) و ولتاژهای 6 و 10 ولت بر روي تشکيل بيوفيلم بررسي شد. براي اين منظور از سویه استاندارد استافيلوکوک اورئوس (ATCC29213S. aureus) و پسودوموناس آئروژينوزا (P. aeruginosa PAO1) استفاده گرديد. ابتدا سوسپانسیون نیم مک فارلند تهیه و سپس توانایی تولید بیوفیلم نمونه‌هاي آزمون و شاهد با استفاده از روش میکروتیترپلیت، بررسي و نتايج آنها با هم مقایسه گردید. نتایج: جريان متناوب با فرکانس‌های بالا (20 مگاهرتز) و ولتاژهای پايين (6 ولت) بر روی استافيلوکوک اورئوس، اثر بازدارندگیقوي تا متوسط و بر روي پسودوموناس آئروژينوزا، اثر بازدارندگي متوسط تا ضعيف داشت. نتيجه‌گيري: داده‌هاي اين مطالعه استفاده از آثار مثبت تحريك الكتريكي با جريان متناوب را در پيشگيري و رفع عفونت‌هاي باكتريايي پيشنهاد نموده، به‌نظر می‌رسد كه با جستجوی الگوهاي تحريك الکتريکي متناوب جدید بتوان از اين خصوصيت به‌عنوان یک مکمل برای حذف بيوفيلم و افزایش کارآیی آنتی‌بیوتیک‌ها و مواد ضدعفونی در برابر بیوفیلم استفاده كرد.

Title: اثر کوارستین روی نقایص لوله عصبی و ناهنجاری‌های اسکلتی ناشی از والپروات سدیم در جنین‌های موش صحرایی

http://knh.shmu.ac.ir/index.php/site/article/view/711

مقدمه: والپروات سديم، نمك سديمي والپروئيك اسيد و يك داروي ضد تشنج می‌باشد كه در درمان صرع به‌كار می‌رود و آثار تراتوژنيك آن در مطالعات متعددي به اثبات رسيده است. گزارش‌های متعددی وجود دارد که آثار تراتوژنیکوالپرواتسديم به‌وسیله داروهاي آنتی‌اکسیدان يا تحریک سیستم ایمنی مادری کاهش می‌یابد. به‌نظر مي‌رسد كوارستين داراي خاصيت آنتی‌اکسیدانی باشد. هدف مطالعه حاضر، بررسی نقش محافظتی كوارستين روی ناهنجاری های ناشی از والپروات سديم در جنین موش صحرايي بوده است. مواد و روش‌ها: اين مطالعه در 6 گروه از موش‌های صحرايي آبستن (در هر گروه 6 سر) انجام شد. به گروه اول (گروه کنترل) نرمال سالین به‌صورت داخل صفاقی تجويز شد و به گروه دوم والپروات سديم با دوز 300 میلی‌گرم به ازای هر کیلوگرم وزن بدن تجویز شد. به گروه سوم و چهارم به‌ترتيب كوارستين با دوز 75 و 200 میلی‌گرم به ازای هر کیلوگرم وزن بدن تجویز شد. گروه پنجم والپروات سديم همراه با كوارستين با دوز 75 میلی‌گرم به ازای هر کیلوگرم وزن بدن و گروه ششم، والپروات سديم همراه با كوارستين با دوز 200 میلی‌گرم به ازای هر کیلوگرم وزن بدن را به روش داخل صفاقی دریافت نمودند. در روز بیستم آبستنی، جنین‌ها از رحم خارج شدند و پس از تعیین طول و وزن جنین‌ها، مورد رنگ‌آمیزی آلیزارین قرمز-آلسین آبی قرار گرفته و ناهنجاری‌ها به‌وسیله استریومیکروسکوپ مشخص گردیدند. نتايج: درصد وقوع شکاف کام، مهره ‌شکاف‌دار و اگزنسفالي در گروه دریافت‌کننده والپروات سديم به‌تنهایی به‌ترتيب 7/17%،20% و20% بود که به‌وسیله كوارستين با دوز 75 میلی‌گرم به ازای هر کیلوگرم وزن بدن به 95/13%،62/11% و 95/13% و به‌وسیله كوارستين با دوز 200 میلی‌گرم به ازاي هر کیلوگرم وزن بدن به 11/11% و 11/11% و 11/11% رسید. نتيجه‌گيري: كوارستين می‌تواند وقوع ناهنجاری‌های ناشی از والپروات سديم را کاهش دهد.

Title: مهندسی گلیکوزیلاسیون پروتئین‌های نوترکیب انسانی در سیستم جدید بیانی S2

http://knh.shmu.ac.ir/index.php/site/article/view/714

براي دستيابي به بيان بالاي بسياري از پروتئين‌هاي نوترکیب و پيچيده از سامانه‌هاي بياني حشرات استفاده می‌شود اما حشرات در سنتز محصولات N- گلیکانی شبیه پستانداران ناتوانند. محصولات گلیکوزیلاسیونی در حشرات حاوی واحدهای مانوزی کم یا زیاد انتهایی می‌باشد. دلیل اصلی این ناتوانی، پایین بودن سطح فعالیت تعدادی از آنزیم‌ها شامل β- N، (1-2) استیل گلوکز آمین ترانسفراز I و II، β- (1-4)، گالاکتوزیل ترانسفراز، α، (2-3) و α، (2- 6)- سیالیل ترانسفراز می‌باشد. از طرفی یک هگزوآمیدیناز که سبب حذف N- استیل گلوکزآمین از انتهای محصول گلیکانی شده و مانع از اتصال گالاکتوز و اسید سیالیک به محصولات گلیکانی می‌شود، در حشرات کشف شده است. لذا می‌توان حشرات را در جهت تولید محصولات گلیکوزیلاسیونی مشابه پستانداران مهندسی کرد و عوامل مهار‌کننده سنتز محصولات سیالیل‌دار و گالاکتوز‌دار را از پیش‌رو برداشت. لذا در این مقاله مروری سیستمیک به مسیرهای آنزیمی گلیکوزیلاسیون در پستانداران و حشرات و مهندسی مسیرهای ممکن گلیکوزیلاسیون در سلول‌های حشره دروزوفیلایی S2، پرداخته شده است.

Title: بررسی حذف سرب از محلول‌های آبی با استفاده از جاذب‌های جدید تهیه شده از پوست پسته اصلاح شده

http://knh.shmu.ac.ir/index.php/site/article/view/775

مقدمه: فلزات سنگین نظیر سرب از آلاینده‌های مهم زیست‌محیطی محسوب مي‌شوند. هدف این مطالعه بررسی میزان حذف آلودگی سرب از محلول‌های آبی با جاذب‌های پوست پسته اصلاح شده بود. مواد و روش‌ها: دراین مطالعه قابلیت حذف سرب از محیط‌های آبی توسط پوست پسته اصلاح شده مورد بررسی قرار گرفت. پوست پسته از باغات پسته شهرستان دامغان تهیه گردید. برای تهیه جاذب‌ها، پوست‌های پسته به‌طور جداگانه با محلول‌های NaoH (4/0 مول در لیتر) و HNo3 (4/0 مول در لیتر) و آب مقطر دو بار تقطیر اصلاح شده و قدرت جذب جاذب‌های تهیه شده با یکدیگر مقایسه گردیدند. جذب سرب توسط جاذب‌های پوست پسته اصلاح شده در سیستم ناپیوسته مطالعه شد. بعد از بررسی pH بهینه در روش تک عاملی، آزمایش‌های جذب با روش تاگوچی طبق آرایه L16انجام گرفت و اثر چهار عامل مؤثر بر جذب شامل: غلظت‌های اولیه سرب (5، 10، 15 و 20 میلی‌گرم در لیتر)، pH‌‌های (4، 5، 6 و 7)، جرم‌های مختلف جاذب (5/0، 1، 5/1 و 2 گرم در لیتر) و زمان تماس‌های متفاوت (30، 60، 90 و 120 دقیقه) در چهار سطح بررسی شد. نتايج: مطالعه حاضر نشان داد که حداکثر راندمان جذب سرب توسط پوست پسته اصلاح شده با محلول بازی بوده که در شرایط pH برابر 4، غلظت اولیه 5 میلی‌گرم در لیتر سرب، زمان تماس 90 دقیقه و جرم جاذب 5/0 گرم در لیتر، برابر با 25/91 درصد بوده است. در حالی‌که در شرایط برابر، حداکثر راندمان جذب توسط پوست پسته اصلاح شده اسیدی برابر 05/89 درصد بوده است. همچنین در همین شرایط حداکثر ظرفیت جذب توسط پوست پسته اصلاح شده بازی و اسیدی به‌ترتیب برابر با 5/36 و 4/33 میلی‌گرم بر گرم بوده است. نتیجه‌گیری: براساس نتایج این مطالعه، پوست پسته اصلاح شده قلیایی دارای قابلیت بیشتری برای حذف سرب از محلول‌های آبی می‌باشد و مي‌تواند به‌عنوان یک جاذب ارزان در حذف سرب از محلول‌های آبی مورد توجه قرار گیرد.

Title: اثر عصاره هيدرواتانولي ميوه گیاه زرشک (Berberis Vulgaris L.) بر بيليروبين و آنزیم‌های کبدی سرم خون موش‌های صحرایی نر كلستاتيك

http://knh.shmu.ac.ir/index.php/site/article/view/783

مقدمه: اختلالات كبدي اغلب با افزايش بيليروبين خون و آنزيم‌هاي كبدي در پلاسما همراه هستند. در این بررسی اثر پايین‌آورندگی بيليروبين خون گیاه زرشک (Berberis vulgaris) مورد بررسی قرار گرفت. مواد و روش‌ها: در این مطالعه تجربی تعداد 42 سرموش صحرایی نر ویستار با محدوده وزنی220-250 گرم به‌طور تصافی در شش گروه كنترل، شم، كلستاز، كلستاتيك درمان شده با عصاره زرشك (دوزهاي 300 و 600 میلی‌گرم بر کیلوگرم، روزانه به‌مدت یک هفته) و كلستاتيك درمان شده با داروي فنوباربيتال (15 میلی‌گرم بر کیلوگرم) تقسيم شدند. موش‌ها با عمل لاپاراتومي و بستن مجراي صفراوي مشترك به كلستاز مبتلا شدند. بعد از سه روز در موش‌ها كلستاز ايجاد و هايپربيليروبينمي به وقوع پيوست. در پایان آزمایش، نمونه‌هاي خون به‌طور مستقيم از قلب موش‌ها تهيه و سرم آنها جدا و بيليروبين كل، مستقيم و غيرمستقم و آنزيم‌هاي كبدي توسط کیت الايزا اندازه‌گيري شدند. اختلاف داده‌ها با 05/0P< معني‌دار در نظر گرفته شد. نتایج: اين بررسي نشان داد كه موش‌هاي كلستاتيك درمان شده با عصاره زرشك به‌مدت يك هفته از كاهش معني‌دار بيليروبين خون و آنزيم‌هاي کبدی نسبت‌به گروه‌هاي كنترل و كلستاتيك درمان شده با داروي فنوباربيتال برخوردار شدند (01/0P<). نتیجه‌گیری: اثر كاهش‌دهندگي بيليروبين خون و آنزيم‌هاي كبدي توسط عصاره زرشك احتمالاً به‌واسطه اثر تركيبات موجود در اين گياه نظير بربرين بر روي كبد و دفع بيشتر بيليروبين از طريق كليه‌ها مي‌باشد.

Title: بررسی شأن اجتماعی و عوامل مرتبط با آن در بیماران مبتلابه نارسایی قلبی

http://knh.shmu.ac.ir/index.php/site/article/view/351

مقدمه: نارسايی قلبی یک مشکل عمده رو به رشد است که نه تنها بیماران بلکه خانواده آنان و شبکه اجتماع را نیز تحت تأثير قرار می‌دهد و باعث کاهش ظرفیت‌های عملکردی بیماران و اختلال در زندگی اجتماعی افراد می‌گردد. پژوهش حاضر با هدف تعیین شأن اجتماعی و عوامل مرتبط با آن در بیماران مبتلابه نارسایی قلبی صورت گرفته است. مواد و روش‌ها: پژوهش حاضر يك مطالعه توصيفي- مقطعی بوده و جامعه مورد مطالعه آن بیماران مبتلابه نارسایی قلبی بستری در بخش قلب بيمارستان‌هاي حضرت رسول (ص)، امام حسین (ع) و شریعتی تهران در سال 1391 بودند. در اين پژوهش، نمونه‏گيري به‌صورت مبتنی بر هدف بوده و تعداد 130 بيمار مبتلابه نارسایی قلبی از ميان جامعه پژوهش انتخاب گرديدند. پرسشنامه اطلاعات دموگرافيك و همچنين پرسشنامه شأن اجتماعی، براساس گفته‌هاي بيمار تكميل گرديد. تجزیه و تحلیل آماری با استفاده از روش‌هاي آماري توصيفي و آزمون‌های آماری پیرسون، آنالیز واریانس یک طرفه و تی مستقل و با کمک نرم‌افزار SPSS نسخه 19 انجام شد. نتایج: میانگین نمره شأن اجتماعی در واحدهای مورد پژوهش 7/0 ± 3/4 محاسبه گردید. بیشترین میانگین نمره مربوط‌به بعد ارتباط و حمایت جامعه و کمترین میانگین نمره مربوط‌به بعد تحمیل شدن به دیگران (اقتصادی) است و آزمون آماری آنالیز واریانس با 05/0P<، تفاوت معني‌داری بین میانگین نمره شأن اجتماعی در دو بعد ذکر شده نشان می‌دهد. آزمون آماری پیرسون، ارتباط منفي معنی‌دار آماری بین دفعات بستری با نمره شأن اجتماعی نشان داد. این آزمون همچنین ارتباط مثبت معنی‌دار آماری بین شدت بیماری (كسر تخليه‌اي بطن چپ) با نمره شأن اجتماعی نشان داد. نتیجه‌گیری: هرچند که نتایج پژوهش حاضر نشان داد که بیماران مبتلابه نارسایی قلبی، از شأن اجتماعی خوبی برخوردار هستند و از ارتباط و حمایت جامعه رضایت دارند ولی از لحاظ اقتصادی احساس تحمیل شدن به دیگران مي‌نمایند. لذا به‌کارگیری حمایت‌های جسمی، روانی و اقتصادی از این بیماران در جهت حفظ و ارتقاء هرچه بیشتر شأن اجتماعی در آنان پیشنهاد می‌شود.
